# Recent Developments of Graphene Oxide-Based Membranes: A Review

**DOI:** 10.3390/membranes7030052

**Published:** 2017-09-12

**Authors:** Jinxia Ma, Dan Ping, Xinfa Dong

**Affiliations:** School of Chemistry and Chemical Engineering, South China University of Technology, Guangzhou 510641, China; 201420120600@mail.scut.edu.cn (J.M.); 201510103698@mail.scut.edu.cn (D.P.)

**Keywords:** membrane, graphene oxide, graphene oxide membrane, separation performance, structural stability

## Abstract

Membrane-based separation technology has attracted great interest in many separation fields due to its advantages of easy-operation, energy-efficiency, easy scale-up, and environmental friendliness. The development of novel membrane materials and membrane structures is an urgent demand to promote membrane-based separation technology. Graphene oxide (GO), as an emerging star nano-building material, has showed great potential in the membrane-based separation field. In this review paper, the latest research progress in GO-based membranes focused on adjusting membrane structure and enhancing their mechanical strength as well as structural stability in aqueous environment is highlighted and discussed in detail. First, we briefly reviewed the preparation and characterization of GO. Then, the preparation method, characterization, and type of GO-based membrane are summarized. Finally, the advancements of GO-based membrane in adjusting membrane structure and enhancing their mechanical strength, as well as structural stability in aqueous environment, are particularly discussed. This review hopefully provides a new avenue for the innovative developments of GO-based membrane in various membrane applications.

## 1. Introduction

In the past few decades, membrane-based separation technology has attracted considerable attention in many separation fields due to its advantages of easy-operation, energy-efficiency, and environmental friendliness [1]. Advanced membranes with superior selectivity and permeability are essential to the development of membrane-based separation technology. Currently, polymeric membrane has governed the entire membrane market, including real-world application and academic research, owing to its advantages of energy-efficiency, easy-operation, low-cost, and inherent simplicity. Nevertheless, restrictions of polymeric membranes still exist for most practical applications, because most of them tend to foul, have low resistance to chlorine, strong acids/alkaline, high temperature and organic solvents, and suffer from aperture shrinkage under high pressure [2]. The strong trade-off relation between membrane selectivity and permeability is a common challenge for all of polymeric membranes [3]. These restrictions have urged membrane scientists to constantly seek new membrane materials and develop novel membrane structures with superior chemical stability, thermal stability, water permeability, as well as high selectivity [4]. Recently, carbon-based materials like carbon nanotubes (CNTs), graphene, and its derivative graphene oxide (GO), have shown notable potential in membrane-based separation fields because of their strong mechanical strength, high resistance to strong acids/alkaline and organic solvents, and easy accessibility [5,6,7,8]. Among them, GO was served as one of the emerging nano-building materials for the fabrication of novel separation membrane owing to its distinct two-dimensional (2D) and single-atomic-thick structure, high mechanical strength, high chemical inertness, nearly frictionless surface, and good flexibility combined with large-scale and cost-effective production in solution [9,10,11].

**Figure 1:**
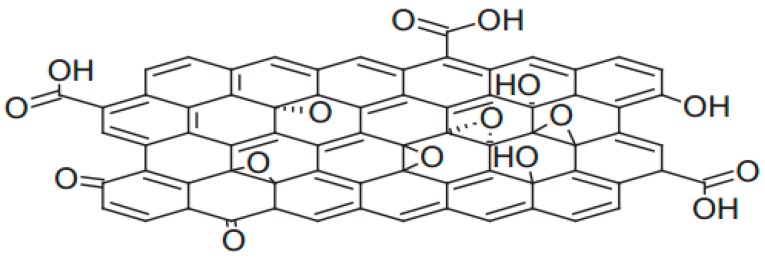
Structure mode of graphene oxide [23]. Copyright 2015 Journal of the Physical Society of Japan.

GO was first synthesized by Brodie [12] in 1859. Subsequently, Staudenmaier [13] and Hummers [14] improved the preparation method in 1898 and 1958, respectively. Afterwards, several modified Hummers’ methods as well as some other new methods were successively developed [15,16,17]. In order to identify the surface morphology and chemical structure of the resultant GO, several characterization techniques are widely employed, such as atomic force microscopy (AFM), scanning electron microscopy (SEM), transmission electron microscopy (TEM), X-ray diffraction (XRD), Raman spectroscopy, X-ray photoelectron spectroscopy (XPS), Fourier-transform infrared spectroscopy (FTIR), thermogravimetric analysis (TGA), and Zeta potential [15,16,18,19]. The resultant GO contains plentiful of oxygenated functional groups—such as hydroxyl, epoxy, and carboxyl—on its basal plane and at its edge [20], as shown in Figure 1. These functional groups endow GO good hydrophilicity and favorable water solubility, which enables a convenient and cost-effective solution process for the preparation of GO-based membrane [21,22]. Additionally, these oxygenated functional groups make GO nanosheets readily to be further surface-modified and the correspondingly functional GO-based composite membranes with preferable separation performance can be obtained.

**Figure 2:**
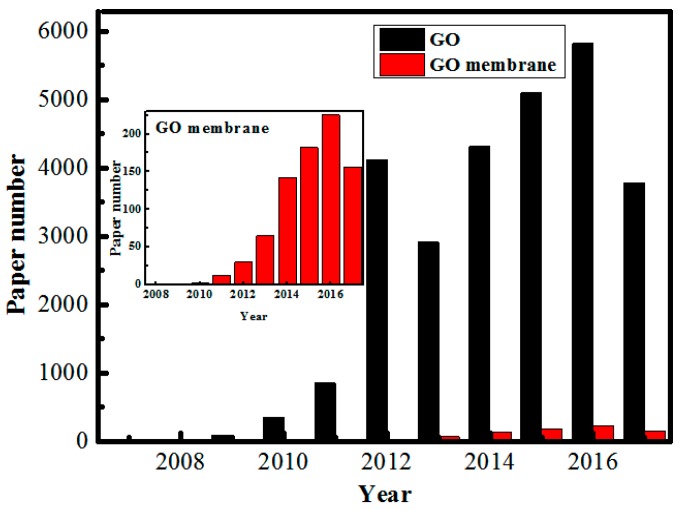
Numbers of publication for GO and GO-based membranes reported between 2008 and 2017 (topic keywords “graphene” and “graphene oxide membrane” searched from web of science), data updated by August, 2017.

Based on these advantages as well as high surface-to-volume ratio structure of GO nanosheets, various GO-based membranes have been widely developed and exhibited great promise in many membrane separation fields such as gas separation [24,25], water purification [26], desalination [27], and pervaporation (PV) [28]. In recent decades, patents and papers (including research articles and review papers) focusing on GO-based membranes are growing exponentially, as shown in Figure 2. Among them, several review papers focused on summarizing the structure, physicochemical property, application, and separation mechanism of GO-based membranes appeared [9,23,29,30,31,32,33,34,35,36,37,38,39]. Based on these research articles, we learned that the structure, mechanical strength, and structural stability of GO-based membrane have significant influence on membrane separation performance.

In this review paper, the latest research progress in GO-based membranes centered on improving membrane structure, mechanical strength as well as structural stability in aqueous solution is highlighted and discussed in more detail. First, we briefly reviewed the preparation and characterization of GO. Then, the preparation method, characterization, and type of GO-based membrane are summarized. Finally, the advancements of GO-based membrane in adjusting membrane structure and enhancing their mechanical strength as well as structural stability in aqueous environment are particularly discussed, in order to promote the development of GO-based membranes in real-world applications.

## 2. Preparation and Characterization of GO

### 2.1. Preparation of GO

**Figure 3:**
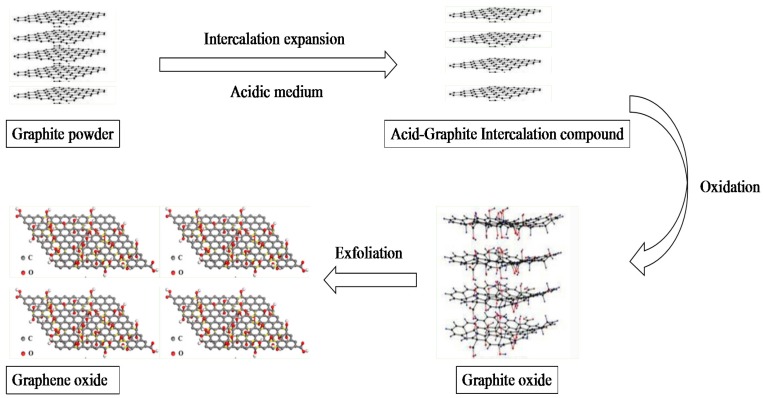
Schematic illustration of GO preparation process.

The synthetic process of GO mainly contains two steps: oxidation of graphite and exfoliation of graphite oxide, as shown in Figure 3. So far, various methods have been reported for the preparation of GO [12,13,14,15,16,17,40]. These methods, as well as their characteristics, are summarized in Table 1.

GO was first synthesized by Brodie in 1859 [12]. In this procedure, graphite was repeatedly oxidized in a fuming nitric acid (HNO_3_) with potassium chlorate (KClO_3_) as the oxidant for three to four days. The extent of oxidation characterized by the C:O ratio was determined to be 2:1. This procedure proved to be time consuming and generated toxic gas (ClO_2_), which was unsafe and harmful to the environment. Nearly 40 years later in 1898, Staudenmaier improved Brodie’s method by adding KClO_3_ in multiple aliquots during the oxidation course and further acidifying the mixture by adding concentrated sulfuric acid (H_2_SO_4_) [13]. This method was more practical and convenient for the production of GO with comparable oxidation degree to Brodie’s method. However, similarly to Brodie’s method, this method also produced toxic gases (ClO_2_, NO*_x_*) and was not environmentally friendly.

In 1937, Hofmann modified Brodie’s method, which substituted fuming HNO_3_ with non-fuming HNO_3_ during the oxidation course [40]. Nearly 20 years after Hofmann, in 1958, a different approach was put forward by Hummers and Offeman, who utilized potassium permanganate (KMnO_4_) as oxidant combined with a hybrid of concentrated H_2_SO_4_ and sodium nitrate (NaNO_3_) [14]. A more highly oxygenated form of GO could be obtained by this method in less than 2 h. As such, this procedure was more efficient and less time consuming compared to the aforementioned methods and widely used in current research. In 1999, Kovtyukhova et al. [17] developed a modified Hummers’ method, which included two oxidation procedures. First, they preoxidized the graphite in a mixing solution of concentrated H_2_SO_4_, K_2_S_2_O_8_, and P_2_O_5_ at 80 °C. Then the mixture was washed and dried at ambient temperature. After that, the mixture was ulteriorly oxidized by Hummers’ method. Compared to Hummers’ method, the oxidization extent of graphite was slightly higher via this method. However, it should be noted that both Hummers’ method and modified Hummers’ method generated toxic gases (NO_2_, N_2_O_4_) and much more attention should be paid to control the reaction temperature during the process.

In order to develop a more convenient and safer method for producing GO, Marcano et al. [16] proposed an improved Hummers’ method in 2010, in which a hybrid of H_2_SO_4_/H_3_PO_4_ with volume ratio of 9:1 was used as the mixed acid and KMnO_4_ was used as the strong oxidant. Compared to Hummers’ method, the improved Hummers’ method was simpler and higher yielding, and generated no toxic gas, making it possible for large-scale production of GO. Nevertheless, all of the Hummers’-related methods faced a common problem, that is the introduction of environmentally hazardous heavy metal Mn^2+^ in the preparation process, and the existence of trace of Mn^2+^ would affect the physicochemical properties of GO. In order to solve this problem, Gao et al. [15] reported a new environmentally-friendly approach, in which the K_2_FeO_4_ was utilized as the strong oxidant to avoid the introduction of heavy metal Mn^2+^. Meanwhile, this procedure was less time consuming (1 h) and enabled the recycle of H_2_SO_4_, which decreased the pollution to environment. They claimed that this green, safe, and highly efficient method was promising for large-scale commercial applications of GO.

As mentioned above, an approach which is highly oxidized, low-cost, safe, simple, and environmentally friendly will provide the possibility for large-scale production of GO. Therefore, continuous efforts are required to achieve this objective. Additionally, it should be noted that the resultant GO produced with different methods differs significantly in structure and physicochemical property, which depends not only the species and dosage of oxidant, but also on the reaction condition and initial graphite source. So the method for GO preparation should be carefully designed in a practical application.

### 2.2. Characterization of GO

In order to verify the successful synthesis of GO and identify its chemical structure, a variety of characterization techniques have been employed. For example, in order to obtain the information of surface morphology and size of GO, SEM, TEM, and AFM are widely used [16,19]. With respect to the chemical composition of GO, quantitative XPS and inductively coupled plasma mass spectrometry (ICP-MS) are usually utilized [15]. Additionally, Raman spectra, XRD, and FTIR spectra are extensively applied to identify the chemical structure of GO [15,16,18]. To obtain more information about GO properties, TGA, and Zeta potential are also employed by researchers to judge its thermal stability and electrochemical property [16,22]. More detailed descriptions about these characterization techniques are summarized in Table 2.

## 3. GO-Based Membranes

### 3.1. Preparation Methods of GO Membranes

Based on stable aqueous dispersity as well as high aspect ratio structure of GO, GO membranes can be easily fabricated via different methods such as filtration-assisted method, casting/coating-assembly method, and layer-by-layer (LbL) assembly method. Additionally, evaporation-assisted method, templating method, shear-induced alignment method, and hybrid method are also applied to prepare GO membranes (Table 3). The different preparation methods for GO membranes will be described in detail as follows.

#### 3.1.1. Filtration-Assisted Method

**Figure 4:**
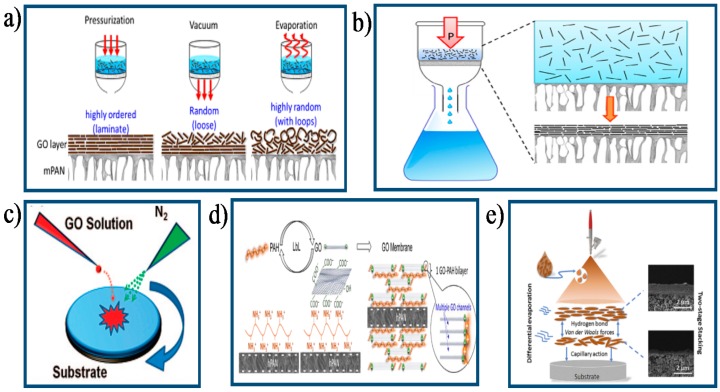
Schematic diagrams of the preparation of GO-based membranes through different approaches: (**a**) Filtration-assisted self-assembly and evaporation-assisted self-assembly technique [43]. Copyright 2015 Elsevier; (**b**) PASA technique [28]. Copyright 2014 Elsevier; (**c**) Modified spin-coating technique [46]. Copyright 2008 American Chemical Society; (**d**) LbL assembly via electrostatic interaction [47]. Copyright 2014 Elsevier; (**e**) Spray-evaporation assembled technique [48]. Copyright 2016 Elsevier.

Filtration-assisted method, including vacuum filtration and pressure-assisted filtration, is a widely used approach to prepare GO membranes at present, especially for the free-standing GO membranes [28,41,42]. Dikin et al. [42] fabricated a free-standing GO membrane by vacuum filtration, in which GO nanosheets were bonded together in a near-parallel way. They reported that the physicochemical property of GO nanosheets did not change during the preparation process. Tsou et al. [43] investigated the influence of GO membrane structure prepared via three distinct self-assembly methods (pressure-, vacuum-, evaporation-assisted technique) on membrane separation performance (Figure 4a). Results showed that the GO membrane obtained via pressure-assisted technique exhibited exceptional PV performance and superior operating stability at a high temperature (70 °C) due to its dense packing and highly ordered laminate structure. In another study, a highly ordered GO/mPAN (modified polyacrylonitrile) composite membrane was prepared via pressure-assisted self-assembly (PASA) technique [28] (Figure 4b). The resultant GO/mPAN composite membrane exhibited excellent PV performance for an isopropyl alcohol (IPA)/water mixture. They reported that the membrane thickness could be readily adjusted by changing the concentration and volume of GO solution.

From above discussion, we can conclude that filtration-assisted method allows reasonable and easy control over the membrane thickness and microstructure, and is a potential route for large-scale preparation of GO membrane.

#### 3.1.2. Casting/Coating-Assisted Method

At present, many GO membranes have been developed based on casting/coating-assembly method, which includes drop-casting [44], dip-coating [45], spaying-coating/casting [6], and spin-coating approach [46]. Park et al. [25] fabricated several layered GO membranes via spin-coating method on a polyethersulfone (PES) substrate and studied their gas separation performance. They reported that high gas separation selectivity could be achieved by controlling gas flow channels through adjusting stacking manner of GO nanosheets. Robinson et al. [46] presented that large-area and ultrathin GO membranes with excellent mechanical property could be obtained by a modified spin-coating method (Figure 4c). In this procedure, dry nitrogen was utilized to accelerate GO solution evaporation, which correspondingly obtained continuous GO membranes with strong interfacial adhesion force between GO nanosheets and substrate surface. Meanwhile, membrane thickness could be controlled on nanometer scales through varying GO concentration in solution or volume of GO suspension. Individual GO nanosheets within GO membranes fabricated by casting/coating-assembly method are strongly held together with hydrogen bonding and Van der Waals force.

#### 3.1.3. Layer-by-Layer Assembly Method

Recently, LbL assembly approach has been attracting great attention for the preparation of GO membranes. An interlayer stabilizing force can be conveniently introduced into laminate GO membranes by electrostatic interaction or covalent bonding through this method [26,49]. Hu et al. [47] have developed a new-type of water purification membrane through this approach (Figure 4d). The negatively charged GO nanosheets were interconnected with positively charged poly (allylamine hydrochloride) (PAH) via electrostatic interaction and then assembled onto a porous PAN support. Results showed that the resultant GO membrane reserved a compact structure in solutions of low ionic strength and showed excellent separation performance. Typically, the membrane thickness can be easily adjusted by changing the number of LbL deposition cycles [47,50].

#### 3.1.4. Other Methods

Apart from aforementioned methods for the preparation of GO membrane, some novel preparation methods such as evaporation-assisted method [48,51], templating-assisted method [52], Langmuir–Blodgett assembly method [53,54], hybrid method [55], and shear-induced alignment method [56] have also been utilized to fabricate GO membrane. Recently, facile engineering of GO membranes was realized via a hybrid approach by Guan et al. [48], in which spray-coating and solvent evaporation-induced assembly technique were included (Figure 4e). They reported that the membrane structure could be finely and conveniently manipulated by adjusting the spraying times and evaporation rate. The resultant GO membranes with ordered and compact structure presented excellent gas separation performance, which exceeded the upper bound of most polymeric membranes. Specifically, this process was less time consuming and more productive compared with filtration method. This study provided a rather facile and productive approach for large-scale preparation of defect-free GO membranes.

Chen et al. [51] fabricated large-area free-standing GO membranes via an evaporation-driven self-assembly method. They reported that the thickness and area of the membrane could be readily adjusted by controlling the evaporating time and the liquid/air interface area. This is a facile and scale-up approach for preparation of GO membrane. Akbari et al. [56] provided a rapid, scalable, and industrially adaptable method, shear-induced alignment method, to produce large-area GO-based membranes by taking advantage of the flow properties of a discotic nematic GO fluid. The resultant membranes had large in-plane stacking order of GO sheets and showed remarkable enhancement in water permeability with comparable or better retention of small organic molecules and ions by molecular sieving and electrostatic repulsion. Meanwhile, the obtained membranes showed good stability in aqueous environments and excellent fouling resistance due to the hydrophilic groups on GO membrane. This shear-alignment processing method is conducive to bridging laboratory curiosity to industrial productivity for GO membranes.

From above description, it can be concluded that various methods have been developed and utilized to fabricate GO-based membrane. Specifically, it should be noted that the structure and separation performance of the resultant GO membranes significantly depended on the fabrication method and corresponding fabrication conditions. Hence, in a specific practical application, a desired GO membrane can be obtained by appropriate preparation method and optimized fabrication conditions.

### 3.2. Characterization of GO Membranes

In order to identify the structure and determine the separation property of GO membranes, various characterization techniques have been exploited, including SEM, TEM, AFM, TGA, contact angle measurement (CA), FTIR, XPS, XRD, Raman spectroscopy, surface zeta potential, and mechanical measurements. Specifically, in order to get the surface characteristics (i.e., membrane uniformity, surface morphology, and surface roughness), cross-sectional morphology and thickness of the synthesized GO membrane, SEM, TEM combined AFM are usually utilized. With respect to the chemical composition and microstructure of membrane, XRD, XPS, and Raman spectroscopy combined with FTIR spectroscopy are most commonly utilized. Moreover, in order to get more insights into the application potential of the resultant GO membranes, surface zeta potential, TGA, CA, and mechanical measurements are further accomplished. Specifically, an experimental characterization technique using an integrated quartz crystal microbalance with dissipation and ellipsometry was proposed by Mi et al. [57]. This characterization technique could accurately quantify the d-spacing of a GO membrane in an aqueous environment and well beyond the typical measurement limit of (~2 nm) of XRD. Detailed information about these characterization techniques are summarized in Table 4.

Despite aforementioned characterization techniques having been extensively utilized for analyzing structure and separation performance of GO membrane, there still remain several challenges for the accurate and deep characterization of the transport passage of GO membrane. For example, there is a lack of in situ characterization technique for evaluating interlayer spacing of GO membrane when the membrane is under operation. Additionally, an appropriate method for calculating the tortuosity of GO membrane has not been developed. Therefore, more efforts are badly needed to achieve better understanding of the separation mechanism of GO membrane.

### 3.3. Types of GO-Based Membranes

Today, there is a blossoming of studies focused on the development of GO-based membrane, including free-standing GO membrane, supported-GO membrane, and GO-modified composite membrane. Specifically, for the free-standing GO membrane, GO membrane is directly used as a separation layer. With respect to the supported-GO membrane, GO membrane is supported by a polymeric or an inorganic substrate with GO layer as the active separation layer. GO-modified composite membrane is referred to the GO-based membranes obtained by directly incorporating GO nanosheets into polymer casting solutions during membrane fabrication process or functionalizing membrane surface by post-coating of the pre-fabricated membrane with GO nanosheets. In this section, the recent advancements in the three GO-based membranes are in detail reviewed. Table 5 summarized their applications and corresponding separation performances.

#### 3.3.1. Free-Standing GO Membranes

At present, a variety of approaches have been employed to fabricate free-standing GO membrane, such as vacuum filtration [59,60], evaporation-driven self-assembly [51], self-assembly process under ambient conditions [61], drop casting [62], and pressurized ultrafiltration (UF) method [63]. Sun et al. [62] developed free-standing GO membranes via a drop-casting method and investigated their water purification performance. They reported that the sodium salts could be effectively separated from the heavy-metals salts and organic contaminants through these free-standing GO membranes. In a different study, free-standing GO thin films were fabricated via a pressurized filtration method and utilized for dehydration of ethanol [63]. The synthesized GO membranes showed excellent separation performance with water permeability of 13,800 Barrer and water/ethanol selectivity of 227. They said that the excellent separation performance was ascribed to the high structural stability and hydrophilicity of the free-standing GO membranes. Recently, Zhao et al. [60] fabricated a free-standing GO-polygorskite (GOP) nanohybrid membrane for oil/water separation. The obtained free-standing nanohybrid GOP membrane presented outstanding separation performance and anti-fouling property for various oil-in-water emulsion systems, which demonstrated the potential application of such GO membranes in wastewater treatment. More detailed information and a comparison of the membrane performance based on these descriptors are summarized in Table 5.

#### 3.3.2. Supported-GO Membranes

Although free-standing GO membranes have achieved great progresses in membrane separation applications, a GO membrane supported on desired mechanical support for high-pressure application is rather necessary. Additionally, depositing GO layers onto certain polymeric or inorganic membrane surface could also improve the separation performance and antifouling property of pristine membranes. Hung et al. [28] prepared GO-modified PAN composite membranes via PASA technique and studied their PV separation performance for an IPA/water mixture. High permeability and selectivity were obtained by the resultant GO/mPAN composite membrane. They pointed out that the high selectivity of the GO/mPAN composite membranes might be ascribed to the highly ordered, packed laminate and dense structure, which permitted the transport of water but rejected IPA molecules. Recently, a highly permeable and borate cross-linked GO/PES composite membrane was developed via vacuum filtration method and presented efficient carbon capture in separating the CO_2_/CH_4_ mixture [64]. Chu et al. [65] prepared GO-coated PES UF membranes via a simple vacuum filtration process and used the resultant membranes for humic acid (HA) removal. Study results showed that the GO-coated membranes presented approximately 20% higher pure water flux and 3.4 times higher HA rejection than that of the original PES membranes. Meanwhile, they reported that GO sheets were not easily damaged or detached from the PES substrate during filtration or water rinsing due to the strong hydrogen bonding interactions between the sulfone groups on PES and carboxylic groups on GO sheets. Rao et al. [66] fabricated a novel and highly-efficient nanofiltration (NF) membrane via surface decoration of metal-organic framework/GO (IRMOF-3/GO) onto polydopamine (PDA)-coated polysulfone (PSF) substrate and used it for the heavy metal removal from water. Results showed that the resultant NF membrane exhibited a highly-efficient rejection of copper (II) (up to 90%) with a relatively high flux of 31 L/m^2^/bar/h at the pressure of 0.7 MPa and pH 5.0. Additionally, the NF membrane showed excellent stability during the 2000 min NF test. This study provided a promising potential for water purification.

In another study, ceramic hollow fiber supported-GO membranes were prepared via vacuum filtration method by Li et al. [67]. They reported that the synthesized GO membranes showed superior organic solvent NF property. However, such GO membranes were unstable at dry state because GO layers are easily exfoliated from the substrate due to the weak interaction with the support surface. Aiming to solve this problem, several covalent linkers were utilized to strengthen the interfacial adhesion force between GO layers and substrate surface. For example, Goh et al. [68] designed a type of NF-like GO/poly (amide-imide) (PAI) hollow fiber membrane using polyethyleneimine (PEI) as covalent linker. They reported that the resultant novel GO/PAI-PEI composite membranes presented excellent separation performance and great stability for water treatment. Similarly, Jin et al. [69] fabricated a GO/ceramic composite membrane via dip-coating method by modifying the ceramic support surface with silane. The fabricated membranes presented good integrity, continuity, and enhanced stability; and they exhibited superior PV performance for separating water from water/ethanol mixtures. Similar modification technique has been reported by Huang and co-workers [27,70], who prepared highly stable, permselective, and reproducible GO/Al_2_O_3_ and three-dimensional GO framework (GOF)/Al_2_O_3_ composite membrane using PDA and 1, 4-phenylene diisocyanate (PDI) as covalent linker, respectively. The GO nanosheets were strongly bounded onto the support surface due to the great adhesive abilities of PDA and PDI. Both of the modified GO/Al_2_O_3_ and GOF/Al_2_O_3_ composite membranes presented favorable seawater desalination performance and excellent long-term operation stability with constant ion rejection and water flux for 3.5 wt % seawater. These extraordinary separation performances demonstrated the great potential applications of the covalently cross-linked GO membranes for seawater desalination. Recently, Salehi et al. [71] prepared a novel highly-efficient forward osmosis (FO) membrane by LbL assembly of positively charged chitosan (CS) and negatively charged GO sheets onto a negatively charged sulfonated PES (SPES)-PES substrate via electrostatic interaction. Briefly, the negatively charged SPES-PES substrate was prepared by blending hydrophilic sulfonated PES into PES matrix via phase inversion method. The negatively charged SPES-PES substrate was firstly immersed in the positively charged CS solution and a CS layer was deposited on the substrate surface via electrostatic interaction. Then the CS decorated substrate was soaked in the GO solution and a GO layer was formed on the CS decorated substrate surface via electrostatic interaction and amide bonds formed between the carboxylic groups of GO and amino groups of CS. Study results showed that the membranes obtained by LbL assembly of CS/GO had 2–4 orders of magnitude higher water permeation with a little compromise of the salt rejection than that of the thin film composite (TFC) membrane. Additionally, the LbL assembly of CS/GO membranes had enhanced long-time operation stability due to the amide bonds formed between CS and GO. The CS could be used as an effective crosslinker to crosslink GO sheets onto the negatively charged substrate by electrostatic interactions and to crosslink GO adjacent layers by electrostatic interactions coupled with amide bonds formed between CS and GO, which could significantly enhance interfacial compatibility between GO active layer and substrate as well as inter-layer bonding force within GO layers. The more detailed information and comparison of the membrane performance based on these works are summarized in Table 5.

#### 3.3.3. GO-Modified Composite Membranes

Apart from the two types of GO-based membrane described above, in which GO nanosheets were directly used as the active separation layers, researchers also focused on modifying polymeric membranes with GO nanosheets via different methods. By now, various GO-modified polymeric composite membranes have been developed and presented improved water permeability, selectivity, and anti-microbial performances [72,73,74]. Specifically, based on the modified methods of GO, two strategies have been developed to modify the polymeric membranes. For the first method, the GO nanosheets were directly incorporated into polymeric casting solutions during membrane fabrication process [73,75,76,77,78,79,80,81,82]. For the other one, GO nanosheets were utilized to functionalize polymeric membranes via surface modification technique [83,84,85].

The first approach has been employed by several researchers. Lee et al. [73] fabricated GO-incorporated PSF membrane bioreactors (MBRs) via phase-inversion method and investigated their performance. Results showed that the resultant MBRs exhibited excellent anti-fouling performance and a five-fold increase in the time between scheduled chemical cleaning. A new-type of PES composite matrix membrane embedded GO nanosheets was developed via phase-inversion method by Zinadini et al. [86]. The obtained composite membranes with wider finger-like pore structure and superior hydrophilicity compared to the pristine PES membranes exhibited improved water permeability and fouling resistance. In the same way, Ding et al. [87] also synthesized solvent resistant NF GO-embedding PEI/PAN membranes. Briefly, the PAN substrate was first modified with dopamine to strengthen the interfacial bonding force between the GO-embedding PEI layer and PAN substrate. They found that the GO nanosheets were horizontally-aligned within PEI matrix and provided particular transport channels for small-sized molecules whereas rejecting large-sized molecules. With such a unique membrane structure, enhanced solute rejection and solute flux were achieved. Recently, a new-type of thin film nanocomposite (TFN) membrane was prepared by Lai et al. [72], who incorporated different quantities of GO into PSF substrate. The obtained TFN membrane presented higher permeability and selectivity compared with the pristine TFC membrane. The 0.3 wt % GO incorporated TFN membrane exhibited highest water flux (353.5 L/m^2^/bar/h) with superior rejections for Na_2_SO_4_ (95.2%), MgSO_4_ (91.1%), MgCl_2_ (62.1%), and NaCl (59.5%). More critically, the resultant TFN membrane overcame the trade-off effect between permeability and selectivity owing to the improved hydrophilicity and surface negativity after GO incorporating. Zhang et al. [76] fabricated shear-aligned GO laminate/polyethylene oxide-polyamide block copolymer (Pebax) ultrathin composite hollow fiber membranes by dispersing GO into Pebax solution via a facile dip-coating approach. Study results showed that the introduction of the aligned GO laminates into the composite membrane remarkably improved the CO_2_ permeance (up to 90%) of the original Pebax membrane without compromising the CO_2_/N_2_ selectivity. Specifically, the incorporation of GO strikingly improved the Young’s modulus of the composite membrane, which contributed to the mechanical strength of GO and the good interfacial compatibility between GO and Pebax polymers. Additionally, the flexibility and mechanical properties of the resultant composite membrane were significantly enhanced, which were preferable for large-scale manufacture of the shear-aligned GO laminate/Pebax composite hollow fiber membrane.

Perreault et al. [83] utilized the second method to improve the property of TFC polyamide (PA) membrane via a simple GO surface functionalization. Briefly, GO was strongly bounded to the membrane surface through the amide coupling between carboxyl groups of GO and carboxyl groups of the PA active layer. The post-coating surface functionalization strategy allowed GO nanosheets presenting at the membrane surface and conveniently inactivated bacteria. In addition, this method could remarkably reduce the quantity of GO required for the functionalization and correspondingly lowered the cost. Study results showed that the functionalized-TFC PA membranes exhibited excellent antimicrobial property with bacteria directly contacting the membrane surface, which resulted in 65% bacterial inactivation after 1 h. These results demonstrated that the surface functionalization of TFC membranes by GO was a potential route for the design of novel antimicrobial membranes. Recently, Zhang et al. [84] fabricated a GO/aminated PAN (GO/APAN) fiber hierarchical-structured membrane by controlled assembly of GO sheets on the surface of APAN fibers and the gap between fibers, and used them for microfiltration (MF) of oil–water emulsion. Study results showed that the resultant membrane had ultra-high water flux (~10,000 L/m^2^/h) due to the superhydrophilicity and large porosity of GO/APAN membrane. Moreover, the GO/APAN membrane also presented preferable rejection ratio (≥98%) and excellent fouling resistance due to the smaller GO sheets modified on the APAN fibers and larger GO sheets assembled on the gap between fibers. More importantly, the GO/APAN membrane exhibited transnormal stability in separating oil–water emulsion with a broad pH range or high-concentration salt. These results indicated that the novel GO/APAN membrane was promising for practical applications in treating oily wastewater. A similar study was also proposed by Zhang et al. [85] with analogous results. More detailed information and a comparison of the membrane performance based on these studies are summarized in Table 5.

From above discussion, we can conclude that different kinds of GO-based membranes could be fabricated via various preparation methods, which showed superior separation performances in various applications including water purification, wastewater treatment, gas separation, and PV. Based on the discussion and analysis of the presented works, the GO-based membranes had promising potentials in real-world applications by selecting the appropriate preparation method. For example, a rapid, scalable, and industrially adaptable method—shear-induced alignment method—was proposed by Akbari et al. [56] to produce large-area GO-based membranes. The resultant membranes not only had enhanced water permeability but also showed excellent stability and fouling resistance in aqueous environments. This method was conductive to bridging laboratory curiosity to industrial productivity for GO membranes. In an another study, a hybrid approach including spray-coating and solvent evaporation-induced assembly technique was proposed by Guan et al. [48] to engineer GO membranes. The membrane structure could be finely and conveniently manipulated through this method with less time consuming and more productive compared with filtration method. The resultant GO membrane exhibited excellent gas separation. They reported that this method provided a rather facile and productive approach for large-scale preparation of defect-free GO membranes. While the aforementioned methods were predicted to be the scalable and industrially adaptable methods for preparing GO membrane, many more efforts should be taken to prepare highly-efficient GO-based membranes with enhanced separation performance and long-term operation stability to realize the real-world application of GO membranes.

## 4. Enhanced Separation Performance of GO Membrane

Although GO membrane has shown good permeability and selectivity in research experiments, there significant effort is still needed to enhance its separation performance to realize its real-world application and meet industrial demands [88]. So far, several strategies have been put forward to develop high-efficiency GO membranes with improved separation performance for the requirements of specific applications. Herein, according to the different modified ways and interaction between modifying agent and GO sheets, two approaches are introduced: physical approach and chemical approach. Additionally, several other unique methods were also developed by researchers for enhancing the separation property of GO membranes. All of these approaches will be reviewed and discussed in detail as follows.

### 4.1. Physical Approach for Improving Separation Performance of GO Membrane

For the physical approaches, separation property of GO membrane can be improved by controlling GO nanosheets size [44,89,90] and GO membrane thickness [91]; changing water pH [22]; controlling the fabrication condition [92]; intercalating nanoscale materials such as carbon dots (CDs) [93], single-walled carbon nanotube (SWCNT) [94], palygorskite nanorods (PGS) [60], metal–organic framework (MOF) [95] into laminar GO membranes; or incorporating surfactants such as cetyltrimethylammonium bromide (C16TAB) into laminated GO membrane. Shen et al. [44] fabricated GO-polyether block amide (PEBA) composite matrix membranes with different lateral size of GO sheets for CO_2_ separation. Results showed that the membrane microstructure, physicochemical property, and gas separation performance were greatly influenced by the lateral size of GO sheets. Coleman et al. [91] reported a study of two charge-equivalent ruthenium complex ions ((Ru(bpy)_3_^2+^ and Ru(phen)_3_^2+^)) transporting through GO membranes with different thicknesses. Despite only a sub-angstrom size difference between the two ions, their diffusion rates through the GO membranes were markedly distinct. Their analysis suggested that the flow rate ratio of Ru(phen)_3_^2+^ to Ru(bpy)_3_^2+^ declined significantly with the increasing of membrane thickness. They pointed out that for the relatively thin GO membranes, ion transport was mainly accelerated by large pores (>1.75 nm in diameter). Whereas, for the thick membranes, inter-layer spacing formed between adjacent GO sheets dominated only. Huang et al. [22] demonstrated that separation performance of small molecules through GO membranes could be readily controlled by tuning the nanochannels within GO membranes by adjusting the water pH. They reported that at low pH (2–6), the pore size of membrane remarkably decreased with the decreasing water pH because of the increased electrostatic repulsion force between adjacent GO sheets, which correspondingly reduced permeability and increased selectivity of GO membranes. At pH ≤ 2, the GO membranes were nearly impermeable to water. When the pH was in the range of 6–8, the pore size of nanochannels almost kept constant owing to the unchangeable negative charges on GO sheets. Consequently, the permeability and selectivity of GO membranes had no remarkable change. When the pH exceeded 9, the negative charges on GO sheets still almost unchanged, but the ionic screening effect became significant due to the increasing ion concentration in water. This shrank the inter-layer spacing of GO sheets and correspondingly resulted in a reduction of permeability and an increase of rejection rate. Recently, Xu et al. [92] reported that the interlayer nanostructure of ultrathin GO membranes could be easily tuned by simply controlling single layer GO (SLGO) deposition rate. Study results showed that the GO membranes fabricated by slow deposition of SLGO sheets had 2.5–4 times higher water permeation rate and 1.8–4 times higher salt rejection than that of the membranes prepared by fast deposition. This enhancement could be attributed to the structure formed by slow deposition of SLGO sheets, which was more thermodynamically favorable and accelerated fast water permeation. This study demonstrated that the trade-off between water flux and selectivity of GO membranes could be broken by self-assembly of SLGO via simple deposition rate control.

**Figure 5:**
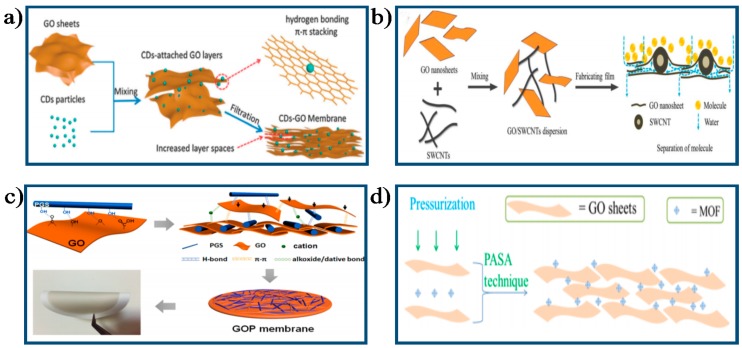
Schematic diagram of the process tuned the structure of GO membrane by physical method: (**a**) The fabrication process of CD–GO membranes [93]. Copyright 2014 Royal Society of Chemistry; (**b**) The preparation process of the SWCNT-intercalated GO ultrathin membrane [94]. Copyright 2015 Royal Society of Chemistry; (**c**) The fabrication process of the GOP membranes [60]. Copyright 2016 American Chemistry Society; (**d**) The fabrication process of the MOF@GO Membranes via PASA technique [95]. Copyright 2017 American Chemistry Society.

**Figure 6:**
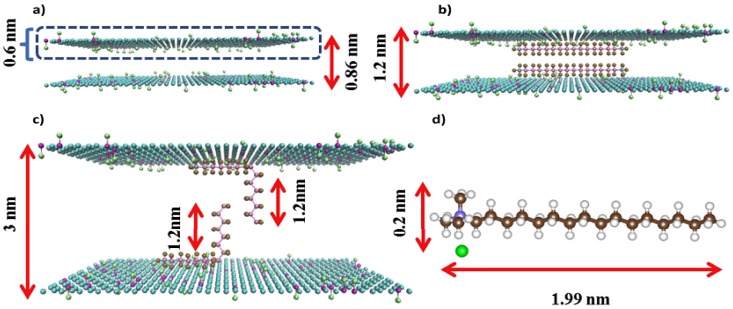
Schematic illustration of possible arrangement of C16TAB within GO membranes: (**a**) The pure GO membrane; (**b**) Two C16TAB paralleled to GO plane; (**c**) Two C16TAB perpendicularly arranged to GO laminate plane; (**d**) Molecular structure of C16TAB with C1–N chain length about 1.99 nm [96]. Copyright 2017 Elsevier.

Wang et al. [93] fabricated GO membranes with adjustable permeability by incorporating controllable sized CDs into the interspace between GO layers (Figure 5a) and studied their filtration performance. They found that the porosities of the CD-embedding GO membranes increased by 42–171% due to the enlarged nanochannels within GO layers compared with those control GO membranes, which remarkably improved membrane permeability coupled with high-efficiency removal rates of organic pollutants. Specifically, the stability of the CDs-embedding GO membranes was also enhanced due to the more compatible integration of the two materials. Gao et al. [94] prepared ultrathin GO membranes with expanded nanochannels by intercalating SWCNT into GO layers (Figure 5b) and evaluated their separation performance. Results showed that the SWCNT-intercalated GO membranes presented higher permeability than original GO membranes with similar rejection rates for nanoscale molecules and particles. In another study, a free-standing GO nanohybrid membrane was developed by Zhao et al. [60], who tuned the inter-layer spacing of GO membranes by intercalating PGS nanorods into adjacent GO sheets (Figure 5c). Study results showed that the resultant PGS nanorod-intercalated GO (GOP) nanohybrid membrane exhibited a sharp increase in permeate fluxes from 267 L/m^2^/h for GO membrane to 1867 L/m^2^/h for GOP membrane. Moreover, the GOP membranes presented exceptional anti-oil-fouling performance for oil-in-water emulsion system with various conditions. They contributed the enhancement of water permeability, separation efficiency, and anti-fouling properties to the enlarged mass transport channels, increased hydration capacity, and the introduction of hierarchical nanostructures on membrane surfaces after intercalating PGS nanorods into the GO layers. Recently, Ying et al. [95] developed novel MOF-intercalated GO (MOF@GO) composite membranes via PASA filtration method by intercalating superhydrophilic MOFs nanoparticles into GO layers and used the resultant MOF@GO membranes to separate ethyl acetate (EA)/water mixtures (98/2, *w*/*w*) through PV process (Figure 5d). They reported that the MOF@GO membranes presented outstanding water permeation and separation factor for EA/water mixtures. Specifically, a 159% increment of permeate flux and 244% increment of separation factor was obtained for the MOF@GO-0.3 membrane (corresponding MOF loading: 23.08 wt %) compared with pristine GO membrane. Meanwhile, the fabricated MOF@GO membranes presented excellent operation stability with almost unchanged permeability and separation factor during the test period as long as 120 h at 303 K. In a recent work conducted by Lian et al. [96], the C16TAB was applied to increase the inter-layer spacing between adjacent GO layers, as shown in Figure 6. As a result, the inter-layer spacing between adjacent GO sheets increased from 0.86 nm for original GO membrane to 3.0 nm for GO-surfacant membrane (confirmed by the XRD analysis), leading to a drastic increase in permeate fluxes from 1.5 L/m^2^/bar/h for pure GO membrane to 20 L/m^2^/bar/h for GO-surfacant membrane without compromising the rejection for sucrose molecules. The significant increase of inter-layer spacing might be ascribed to the unique arrangement of C16TAB within GO-surfacant membrane, with some portion of the C16TAB molecule (around 1.99 nm length and 0.2 nm width, as seen in Figure 6d) vertically aligned and the rest of horizontally aligned between GO sheets. Specifically, the vertically aligned portions of the C16TAB primarily expanded the inter-layer spacing of GO membranes, as shown in Figure 6c.

### 4.2. Chemical Approach for Improving Separation Performance of GO Membrane

**Figure 7:**
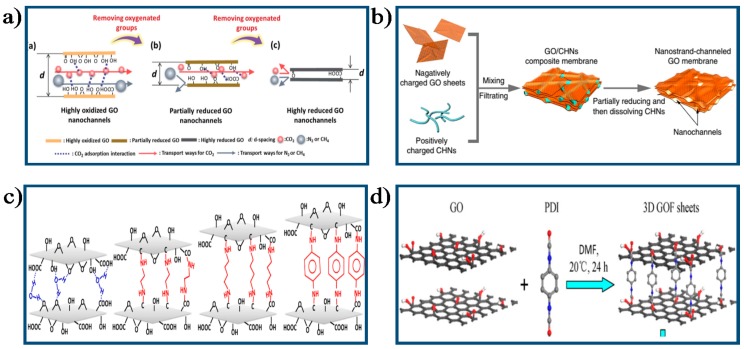
Schematic diagram of the process to tune the structure of GO membrane by chemical method: (**a**) Schematic illustration of the process to tune the microstructure of GO membranes via reduction method [24]. Copyright 2016 Royal Society of Chemistry; (**b**) The fabrication process of NSC–GO membrane [88]. Copyright 2013 Nature Publishing Group; (**c**) The fabrication process of GOF membranes cross-linked with diamine monomers [110]. Copyright 2014 American Chemistry Society; (**d**) The fabrication process of GOF membrane [27]. Copyright 2016 Elsevier.

For the chemical approaches, separation property of GO membranes can be adjusted by changing GO membranes structure through reducing GO membrane [6,24,97,98,99,100,101,102,103,104,105,106,107,108,109] or intercalating chemical groups such as copper hydroxide nanostrands (CHNs) [88], diamine monomers [59,110,111], dicarboxylic acids with different chain lengths [112], 1,3,5-benzenetricarbonyl trichloride (TMC) [26], or soft polymer chains such as poly-(vinylpyrrolidone) (PVP) [113] and PDI [27] into GO membranes via covalently cross-linking or electrostatic interactions using the oxygenated functional groups on GO nanosheets, or introducing in-plane nanopores on GO nanosheets [114], or functionalizing GO sheets using one-step carboxylation via nucleophilic substitution reaction [115]. A study conducted by Shen et al. [24] demonstrated a facile thermal annealing method for finely adjusting the transport channel of GO membranes by controllably removing oxygenated functional groups on GO sheets, as shown in Figure 7a. Subnanometer inter-layer spacing within GO membranes could be created by this method and highly-selective gas transport properties can be obtained accordingly. The GO-0.55 membrane (O/C ratio of 0.55) with 0.36 nm inter-layer nanochannels exhibited highest CO_2_/N_2_ separation performance (CO_2_ permeability: 97 Barrer, CO_2_/N_2_ selectivity: 86), transcending the upper-bound for the most advanced membranes. It was further demonstrated that the size of inter-layer nanochannels within GO membranes could be finely and effectively regulated by controlling the oxygenated groups on GO sheets via chemical reduction.

Huang et al. [88] fabricated nanostrand-challenged GO (NSC–GO) membranes by incorporating positively CHNs (diameter around 2.5 nm) into GO layers. Briefly, a mixture of CHNs and GO sheets was firstly filtered onto a porous support; then partially reduced with hydrazine for 15 min; lastly, CHNs were removed from the NSC–GO membranes using an acid solution, as shown in Figure 7b. The finally resultant NSC–GO membranes exhibited remarkably enhanced water permeability (10-fold enhancement) compared with that of pristine GO membranes with the similar rejection rate for dye molecules, and showed >100 times higher water permeability than that of the conventional UF membranes with similar rejection. Hung et al. [110] prepared composite GO-Framework (GOF) membranes with varying d-spacing from 10.4 Å to 8.7 Å by utilizing diamine monomers with different structures as cross-linkers, as shown in Figure 7c. The synthesized GOF membrane presented excellent PV performance for a 90 wt % ethanol/water mixture and long-term operation stability due to its short inter-layer spacing and strong chemical bonding between GO layers and diamine monomers. Li et al. [113] put forward a strategy to tune the inter-layer spacing of GO membranes by intercalating water-soluble polymer material PVP into GO layers. Results showed that the permeation rate of Reactive Red X–3B across the PVP-intercalated GO membranes was significantly increased. Recently, Feng et al. [27] utilized the PDI as cross-linker to covalently modify GO nanosheets to form a three-dimensional GO framework (GOF) membrane (Figure 7d). The fabricated 18 μm thick GOF membrane exhibited significantly increased water flux (11.4 53 kg/m^2^/h) and ion rejection (over 99.9%) for 3.5 wt % seawater desalination via PV.

**Figure 8:**
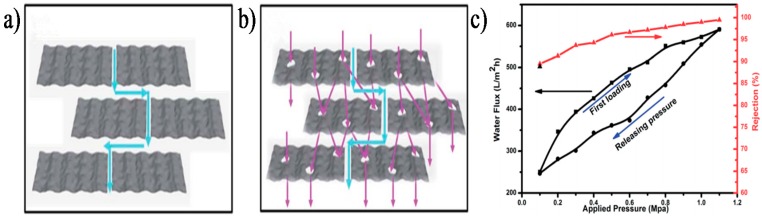
Schematic diagrams of transport path of (**a**) Original GO; (**b**) Mesoporous GO membrane; (**c**) The separation property of mesoporous GO membrane [114]. Copyright 2014 Royal Society of Chemistry.

Ying et al. [114] successfully prepared mesoporous GO sheets by reoxidizing GO with KMnO_4_ and subsequently assembled them into laminar GO membranes for molecule separation. The introduction of in-plane pores not only remarkably diminished the transport path, but also increased the amount of effective channels for water transporting (Figure 8a,b). The mesoporous GO membranes exhibited nearly 2–3-fold enhancement in permeability compared with that of the original GO membranes with the similar rejection rate for small molecules (3 nm). In addition, the mesoporous GO membranes also showed excellent structural stability, which was demonstrated by the pressure loading and releasing process, as shown in Figure 8c. Yuan et al. [115] fabricated GO NF membranes with enhanced desalination performance by functionalizing GO sheets using one-step carboxylation through the nucleophilic substitution reactions between epoxy groups of GO and amino groups of glycine. Study results showed that the GO–COOH membranes presented higher water permeation and salt rejection compared with original GO membranes due to the enhanced surface hydrophilicity, increased water nanochannels, and negativity of GO–COOH membranes. The carboxylation of GO sheets not only enhanced the electrostatic repulsion between adjacent GO–COOH nanosheets but also increased the number of wrinkles on the GO–COOH membranes surface, which resulted in larger nanochannels between GO sheets and correspondingly higher water flux. Additionally, the addition of carboxyl groups on GO sheets increased the negative charge distribution on the GO–COOH membranes surface, which improved the salt rejection of the membranes due to the strengthening electrostatic repulsion between anions and the negatively charged membranes.

### 4.3. Other Approach

**Figure 9:**
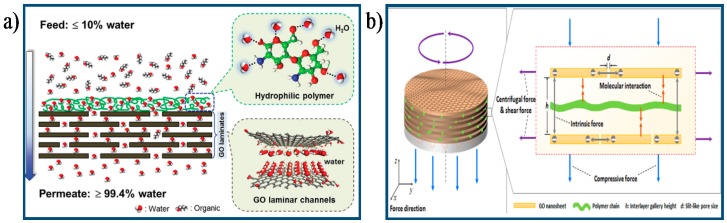
Schematic diagram of the process for improving separation performance of GO membranes: (**a**) Schematic illustration of the integrated GO membrane combined an ultrathin surface water-capturing polymeric layer with GO layers [116]. Copyright 2015 John Wiley and Sons; (**b**) Schematic illustration of the process to tune 2D channels of GO membrane with external force [55]. Copyright 2016 American Chemistry Society.

Apart from the above described strategies for optimizing the separation property of GO membranes, several unique approaches were also developed by researchers for improving the separation performance of GO membranes. Huang et al. [116] found that fast two-dimensional (2D) channels within GO membranes were possibly not fully utilized during the aqueous separation process. Hence, in order to solve the problem to maximize the separation performance of GO membrane, they developed a bio-inspired membrane that combined an ultrathin surface water-capturing polymeric layer (<10 nm) with GO layers, as shown in Figure 9a. Results showed that the integrated membrane showed improved water permeability and the transport channels of GO laminate were fully utilized. Shen and co-workers [55] reported a novel method to accurately regulate the nanostructure of GO-assembled 2D channels, as shown in Figure 9b. The external forces applied in both outside and inside the GO layers effectively overcame the intrinsic electrostatic repulsive force between adjacent GO sheets and correspondingly eliminated non-selective stacking defects. The resultant GO membranes presented 2–3 times higher H_2_ permeability and three-fold improvement in H_2_/CO_2_ selectivity compared with commercial membranes. Recently, Shen et al. [117] proposed a facile method to adjust the inter-layer spacing of GO membrane with solvent green (SG) for improving the NF performance of GO membranes. Results showed that the SG modified GO nanosheets significantly enlarged the inter-layer spacing of the SG@GO composite membrane due to the enhanced static repulsion force between adjacent SG@GO sheets, which resulted in a nearly six-fold enhancement in water flux compared to that of the original GO membranes with the similar rejection rate for dye molecules. In addition, they also found that the resultant SG@GO composite membrane presented excellent pressure resistance ability and long-term operating stability, which contributed to the strong π-π stacking interactions between SG and GO nanosheets.

From above discussion, we can conclude that the separation property of GO membranes could be effectively and successfully improved by different approaches, such as changing GO sheets sizes, or adjusting GO membrane thickness, or intercalating nanomaterials into GO layers, or intercalating chemical groups or soft polymer chains into GO laminar structure, or reducing GO membranes, or introducing in-plane nanopores onto GO sheets. However, there still remain several challenges for tuning the transport channels of GO membrane with these methods. For instance, how to keep the structural integrity of membrane is one big challenge for the reduction treatment on GO membranes. Additionally, special attention should be paid to the compatibility between intercalating material and GO when selecting intercalating nanomaterials. Hence, more efforts should be made to develop highly-efficient GO membranes with improved separation performance.

## 5. Advanced Aqueous Stability and Mechanical Strength of GO Membranes

At present, despite significant advancements in GO-based membranes have been achieved, a few critical challenges in realizing real-world application of GO-based membranes still exist. Specifically, the instability of the inter-layer spacing between adjacent GO nanosheets is a great challenge for utilizing laminar GO membranes as selective aqueous separation barriers, especially for water-related treatment. This is because GO membrane easily disintegrated and redispersed in water over time due to the highly hydrophilic nature of the GO sheets and electrostatic repulsion between the negatively charged GO sheets on hydration , and then the integrity of the laminar GO membranes and inter-layer nanochannels formed by stacking GO sheets would be damaged during aqueous separation process [118,119]. Therefore, it is very much desirable to enhance the structural stability of GO membrane by forming stable bonding between GO nanosheets to realize real-world applications of GO membranes in aqueous environment. Currently, it has been reported that stable GO-based membranes suitable for aqueous system application could be obtained by introducing various cross-linking interactive forces, including electrostatic interactions and covalent bonds between adjacent GO nanosheets or by reducing GO membranes [6,26,34,47,49,98,115,120,121,122,123,124,125,126,127,128,129,130].

**Figure 10:**
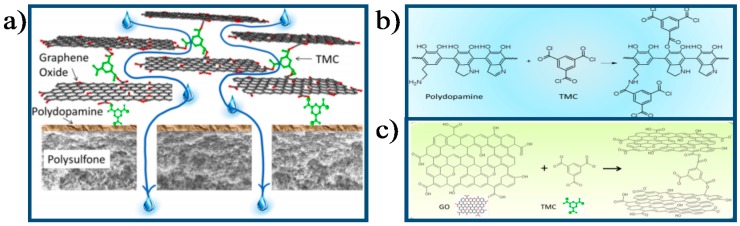
Schematic illustration of (**a**) The forming process of the GO membrane; (**b**) The interaction mechanism between PDA and TMC; (**c**) The interaction mechanism between GO and TMC [26]. Copyright 2013 American Chemistry Society.

Park et al. [122,123] first developed a chemically cross-linking GO membrane using divalent ions and polyallylamine (PAA), respectively. In comparison to the original GO membrane, the modified GO membrane showed significantly enhanced mechanical strength. Using LbL deposition, Mi et al. [26,47] fabricated cross-linked GO membranes using TMC (Figure 10) and positively charged PAH as cross-linkers (Figure 4d), respectively. Results showed that the cross-linked GO membrane exhibited excellent stability for water treatment compared to the pristine GO membrane.

**Figure 11:**
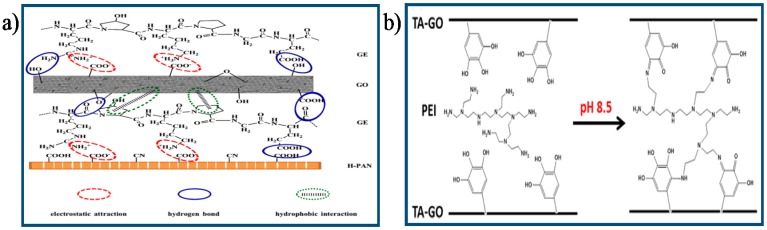
(**a**) Schematic illustration of the interfacial interaction in GO-based hybrid membrane [121] Copyright 2015 Elsevier; (**b**) Schematic illustration of the formation of covalent bonding between adjacent TA–GO sheets [124]. Copyright 2016 Elsevier.

**Figure 12:**
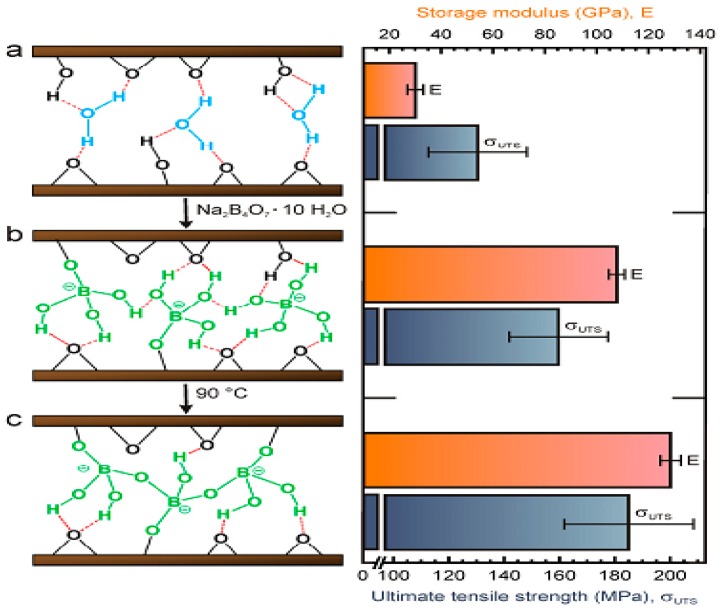
Left: Schematic diagram of the formation of covalent bonding between GO sheets and borate ion. (**a**) Water molecules bound the GO sheets through hydrogen bond; (**b**) Borate anions bound to the GO sheets through covalent bond; (**c**) More covalent bonds formed within the GO sheets after thermal annealing. Right: Mechanical strength of respective films [49]. Copyright 2011 John Wiely and Sons.

A highly stable GO-based ultrathin hybrid membrane was developed by Zhao et al. [121], who utilized gelatin (GE) as the cross-linker to interconnect adjacent GO nanosheets by electrostatic interaction, hydrophobic interaction, and hydrogen bond, as shown in Figure 11a. Enhanced operation stability was obtained for the GE cross-linked GO hybrid membrane used for water/ethanol separation. Recently, a covalently cross-linked GO membrane was developed by Lim et al. [124], who used tannic-acid (TA)-functionalized GO as the membrane material and PEI as cross-linker, as shown in Figure 11b. The resultant cross-linked TA–GO membrane exhibited excellent structural stability in an aqueous environment due to the stable layered structure formed by the cross-linking reaction between TA-GO and PEI. Nguyen et al. [49] fabricated ultra-stiff GO thin films cross-linked GO with borate. They reported that the mechanical strength of the cross-linked GO films obtained by adding 0.94 wt % boron to the GO suspensions was remarkably increased (up to 255% and 20%, respectively) compared to that of the unmodified films. Such significant enhancement was attributed to the strong bonded force between neighboring GO sheets because of the formation of covalent bonds between the hydroxyl groups on GO nanosheets surface and the borate ions (Figure 12). Recently, Liu et al. [127] fabricated highly-aqueous-stable GO membrane by incorporating triethanolamine (TEOA) modified titanate nanowires (TNWs) in GO membrane. They reported that the GO/TEOA–TNWs composite membrane showed significantly improved aqueous stability within even for one month usage due to the strong covalent bonds between the epoxy groups and carboxyl groups on the surface of GO and the N^+^ groups in TEOA. Furthermore, the water flux of the composite membranes was significantly increased due to the intercalation of TNWs between GO sheets, which could introduce plentiful of nanochannels inside the membranes and simultaneously improved surface hydrophilicity of membranes. These results demonstrated that the GO/TEOA–TNWs composite membranes had great potential in the long-term practical water treatment applications. Zhang et al. [128] designed novel GOF membranes using isophorone diisocyanate (IPDI) as a chemical crosslinker for covalent crosslinking of GO nanosheets by a facile vacuum-assisted filtration method. The resultant IPDI–GOF membranes not only presented enhanced structural stability but also showed improved water permeation due to the enlarged nanochannels among GO sheets. The IPDI-GOF membranes exhibited a high water flux of 80 L/m^2^/h under an extremely low pressure (1.0 bar) and excellent removal efficiency for organic dyes molecules (up to 96%). This study provided an approach for enhancing the stability and water permeability of GO membrane which could be applied to real-world water treatment.

Additionally, some studies also showed that reducing GO sheets might also increase the stability of GO membrane by enhancing the π-π interactions between the GO nanosheets. Nevertheless, this would also reduce the water permeation of the membrane because of the shrunken channel distance [6,34,98,115,129]. Yang et al. [129] fabricated PDA-coated reduced GO (PDA–rGO) membranes by chemically reducing GO laminates and then introducing a hydrophilic adhesive PDA layer onto the rGO laminates and used for FO desalination. Study results showed that the resultant PDA–rGO membranes presented excellent aqueous stability and outstanding water flux (36.6 L/m^2^/h) with a high salt rejection rate (92.0%) in FO desalination due to the compacted nanochannels and improved surface hydrophilicity of rGO laminates. They pointed out that the chemical reduction of GO laminates could remarkably increase the salt rejection rate of the membranes by forming highly stable and compacted nanochannels between GO sheets. Moreover, the introduction of a hydrophilic PDA coating onto the rGO laminate surface could further improve the water flux by facilitating the water absorption speed into rGO nanochannels.

Yeh et al. [118] found that the neat GO membranes readily disintegrated in water, but the membranes became stable once they were crosslinked by multivalent cationic metal contaminats (e.g., Al^3+^ and Mn^2+^), which were introduced unintentionally during the synthesis and processing of GO (Supplementary Figures S1 and S2). They contributed remarkably improved membrane stability in water to the unexpected contaminants (i.e., the Al^3+^ in the resultant GO membrane), which acted as crosslinkers and then effectively strengthened the final membrane (Supplementary Figure S3). Meanwhile, they pointed out that significant variability existed for GO membrane stability in water between different modified methods. For example, an around 10% increase in overall stiffness could be observed for GO membrane crosslinked with divalent metal ions [105]. In contrast, in this study even partial Al^3+^ contamination could lead to a more than 340% enhancement in the membrane stiffness. The remarkable variability was attributed to the fact that the ‘unmodified’ GO papers were probably already crosslinked by unintentionally introduced multivalent cationic metal contaminats (e.g., Al^3+^ and Mn^2+^), thus only a modest stiffness difference between unmodified and “crosslinked” GO membrane. That is, for the variability in reported stiffness and stability of GO membranes obtained with different modified methods might be, at least partially, attributed to different degrees of crosslinking by unintentionally introduced contaminants. In order to further identify this point, they removed Al^3+^ from GO (AAO) membranes through ionic exchange with HCl or other monovalent cations such as Na^+^ and Li^+^, after which the membranes readily disintegrated in water (Supplementary Figure S4a,b). XPS detected no Al after the ionic exchange (Supplementary Figure S4c). In addition, they intentionally treated a clean GO (Teflon as the filter disc) membrane with Al^3+^ by utilizing this crosslinking effect, which effectively strengthened the water stability of GO (Teflon) membranes (Supplementary Figure S5). Based on this study, we learned that it is essential for researchers in the field to provide thorough and necessary characterization data for GO (e.g., XPS, XRD in this work) to further identify the potential mechanisms of such phenomena. This finding is very helpful to understand the intrinsic mechanical properties of GO membranes and strengthened mechanism of GO membrane stability in water.

Although the mechanical integrity and structural stability of GO-based membranes could be enhanced in different strategies, more efforts should be taken to prepare highly-efficient GO-based membranes with enhanced separation performance and long-term operation stability for practical applications. At the same time, the intrinsic mechanical properties of GO membranes and strengthened mechanism of GO membrane stability in water should be explored and better understood in more detail.

## 6. Conclusions

In summary, based on the unique single-atomic-thick and two-dimensional structure, together with excellent physicochemical property, GO as an emerging star nano-building material has attracted great interest in the membrane-based separation field. In this review paper, the preparation and characterization of GO were simply summarized. Then we focused on reviewing the preparation method, characterization as well as type of GO-based membrane. Special attention has been paid to the latest advancements of GO-based membrane with respect to the adjustment of membrane structure as well as the enhancement of mechanical strength and structural stability in aqueous environment. An approach which is highly oxidized, low-cost, safe, simple, and environmentally friendly will provide the possibility for massive production of GO. The structure and separation performance of GO membrane significantly depend on the fabrication method and corresponding fabrication conditions. So in a specific practical application, a desired GO membrane can be obtained by employing appropriate preparation method and optimized the fabrication conditions. Despite many characterization techniques having been extensively utilized for analyzing the structure and performance of GO membrane, there still remain several challenges for the accurate and deep characterization of GO membrane. The separation performance of GO membranes could be effectively and successfully improved by different approaches, including physical approach, chemical approach, and some other novel approaches. The mechanical strength and structural stability of GO membrane could be enhanced by different strategies, such as cross-linked GO membrane using different cross-linkers through covalent bonding or electrostatic interaction, or reduced GO membrane through thermal or chemical process to enhance the π-π interactions between the adjacent GO nanosheets. However, several challenges still remain for these strategies. So in order to facilitate the development of GO-based membrane in real-world application, continuous efforts are still required to improve the separation performance and structural stability of GO-based membranes, especially for water-related separation applications.

## Figures and Tables

**Table 1:** Methods for the preparation of GO.

Oxidant	Method	Acid	Reaction Time	Interlayer Spacing	C:O Ratio	Note	Reference
KClO_3_	Brodie	HNO_3_	3–4 days	5.95 Å	2.16	Toxic gas ClO_2_	[12]
	Staudenmaier	HNO_3_, H_2_SO_4_	1–10 days	6.23 Å	1.85	Toxic gas ClO_2_, NO*_x_*	[13]
	Hofmann	HNO_3_, H_2_SO_4_	4 days	–	–	Toxic gas ClO_2_, NO*_x_*	[40]
KMnO_4_	Hummers	NaNO_3_, H_2_SO_4_	≈2 h	6.67 Å	2.25	Toxic gas NO*_x_*, Mn^2+^ in GO	[14]
	Modified Hummers	K_2_S_2_O_8_, P_2_O_5_, H_2_SO_4_	8 h	6.9 Å	2.3	–	[17]
	Improved Hummers	9:1 H_2_SO_4_/H_3_PO_4_	≈12 h	9.3 Å	–	Mn^2+^ in GO	[16]
K_2_FeO_4_	Iron-based green method	H_2_SO_4_	1 h	9.0 Å	2.2	Fe^3+^ in GO	[15]

**Table 2:** Methods for the characterization of GO.

Name	Characterization Method	Characterization Information	Reference
Micromorphology and size of GO	SEM	Lateral size distribution of GO sheets, observe the structural morphology of GO	[15,16]
	TEM	Morphology of GO (wrinkles), monolayer character of GO sheets	[15,16,17,18]
	AFM	Lateral size and thickness of GO sheets	[16,17,18,19]
Thermal stability	TGA	Thermal stability of GO	[15,16]
Chemical structure of GO	XPS	Quantitatively analyze the chemistry composition of GO	[15,16,17]
	ICP-MS	Chemistry composition of GO, identified the metal ion content in GO	[15]
	FTIR	Characteristic bands corresponding to oxygen functional groups, confirmed the successful synthesis of GO	[15,16,17,18]
	XRD	Crystalline structures of the GO nanosheets, inter-sheet distance of GO, confirmed the successful synthesis of GO	[15,16,17,18]
	Raman spectroscopy	Analyze the chemical structure of GO combined with XPS, FTIR, XRD, ICP-MS	[15,16,18]
Electrochemical property	Zeta potential measurement	GO nanosheets are negatively charged over a wide pH range	[22]

**Table 3:** Methods for the preparation of GO membranes.

Method	Description	Note
Filtration-assisted	Vacuum filtration	Good nanoscale control over the membrane thickness; laminar structure of GO membranes is dictated by the filtration force; highly scalable
	Pressure filtration	
Casting/coating-based	Spinning-casting/coating	Nonuniform deposition of GO nanosheets; poor control over the membrane thickness; producing highly continuous GO membranes; highly scalable
	Drop-casting	
	Dip-coating	
	Spray-coating	
	Doctor blade-casting	
LbL assembly	Layer-by-layer assembly	Easily control of the GO layer number, packing, and thickness
Others	Hybrid approach	Easily control of the GO assembly, industrial-scalability, rapid throughput.
	Evaporation-assembled method	Scale-up, easily control of the membrane thickness and size
	Templating method	–
	Langmuir–Blodgett (LB) assembly	Producing highly uniform, close-packed monolayered GO membrane
	Shear-alignment method	Scale-up, industrial-scalability, producing large-area GO membrane, rapid throughput

**Table 4:** Methods for the characterization of GO membrane.

Characterization Method	Characterization Information	Reference
Surface Zeta potential	Identified the surface charges of membrane	[22]
Stress–strain curves	Mechanical stability of the membrane, tensile strength, Young’s modulus	[22]
SEM	Surface morphology and cross-section structure	[26]
AFM	Surface roughness of membrane, membrane uniformity	[26]
CA	Surface hydrophilic or hydrophobic property of membrane	[27]
FTIR	Chemical structure of membrane, surface functional groups of membrane	[48]
XPS	Quantitatively analyze the elemental compositions of membrane	[48]
Raman spectroscopy	Identified the existence of GO in composite membrane	[48]
TGA	Thermal stability of membrane	[49]
TEM	Surface morphology and cross-section structure	[53]
XRD	Crystalline structures, d-spacing of membrane	[58]
Integrated quartz crystal microbalance with dissipation and ellipsometry	Accurately measure the d-spacing of GO membranes in an aqueous environment	[57]

**Table 5:** Application and separation performance of GO-based membranes.

Types of GO Membrane	Name of GO Membrane	Fabrication Method	Application	Membrane Performance	Reference
Free-standing	GO membrane	Flow-directed self-assembly	–	Elastic modulus: 32 GPa Tensile strength: 70.7 MPa	[42]
	GO membrane	Evaporation-driven LbL self-assembly	–	Elastic modulus: 12.7 GPa Tensile strength: 67.7 MPa	[51]
	Cross-linked GO membrane	Vacuum filtration	Ion dialysis separation	Elastic modulus: 10.5402 GPa K^+^/Mg^2+^ selectivity factor: 7.15	[59]
	GOP nanohybrid membrane	Vacuum filtration	Oil/water separation	Water flux: 1869 L/m^2^/h Superior anti-oil-fouling	[60]
	GO membrane	Self-assembly under ambient condition	–	Tensile strength: 46.20 MPa Elongation: 1.29% Young’s modulus: 5.08 GPa	[61]
	GO membrane	Drop-casting	Ion penetration	Entirely blocked heavy-metal salt (e.g., copper sulfate) and organic contaminants (rhodamine B); low rejection of sodium salts	[62]
	GO membrane	Pressurized ultrafiltration	Dehydration of 85 wt % ethanol	Water permeability: 13,800 Barrer Selectivity: 227	[63]
Supported	GO/PES	Spin-casting	Gas separation	CO_2_ permeability: 8500 Barrer CO_2_/N_2_ selectivity: 20	[25]
	GOF/Al_2_O_3_	Vacuum filtration	3.5 wt % seawater desalination	Water flux: 11.4 kg/m^2^/h Ion rejection: >99.9%	[27]
	GO/mPAN	Pressure-assisted	Pervaporation of a 70 wt % IPA/water mixture	Permeation flux: 4137 g/m^2^/h Separation factor: 1164	[28]
		self-assembly			
	GO/PAN	LbL assembly	Water purification	Water flux: 2.1–5.8 L/m^2^/h Sucrose rejection: 99%	[47]
	GO/Nylon	Shear-alignment method	Water treatment	Water permeability: 71 ± 5 L/m^2^/bar/h Rejection: organic probe molecules (hydrated radius >5 Å): >90% Monovalent and divalent salts: 30–40%	[56]
	GO/PES	Vacuum filtration	Gas separation	CO_2_ permeance: 650 GPU CO_2_/CH_4_ selectivity: 75	[64]
	GO/PES	Vacuum filtration	Humic acid removal	Rejection: 85.3–93.9% Superior antifouling capability	[65]
	IRMOF-3/GO/PDA-PSF	Dip-coating	Heavy-metal removal	Water flux: 31 L/m^2^/h Copper(II) rejection: 90%	[66]
	GO/ceramic	Dip-coating	Pervaporation separation of water/ethanol mixtures	Total flux: 461.86 g/m^2^/h Water recovery: 39.92 wt %	[69]
	GO/Al_2_O_3_	Vacuum filtration	3.5 wt % seawater desalination	Water flux: 48.4 kg/m^2^/h Ion rejection: ≥99.7%	[70]
GO-modified	GO/PSF	Phase inversion	Water purification	water flux: 353.5 L/m^2^/bar/h Rejection: Na_2_SO_4_ (95.2%); MgSO_4_ (91.1%); NaCl (59.5%)	[72]
	GO/PSF	Phase inversion	Water treatment	Water flux: 450 L/m^2^/h BSA rejection: 99%	[73]
	GO/PESc	LbL	Water treatment	Water flux: 7.1 kg/m^2^/MPa/h Rejection: Mg^2+^ (92.6%) Na^+^ (43.2%)	[75]
		Self-assembly			
	Pebax/GO/PVDF	Dip-coating	Gas separation	N_2_ permeance: 9.6 GPU CO_2_ permeance: 413.3 GPU CO_2_/N_2_ seletivity: 43.2	[76]
	GO/H-PAN	Electrospinning	Oil/water separation	Water flux: 3500 L/m^2^/h Rejection ratio: 99% Superior anti-oil-fouling	[83]
	GO/APAN	–	Oil/water separation	Water flux: 10,000 L/m^2^/h Rejection ratio: ≥98% Superior anti-oil-fouling	[84]
	GO/PEI/DPAN	Dip-coating	Solvent resistant NF	Ethanol flux: 10.8 L/m^2^/h Acetone flux: 15.7 L/m^2^/h Ethyl acetate flux: 12.9 L/m^2^/h *n*-heptane flux: 3.1 L/m^2^/h PEG(Mw 200) rejection 96.8%	[85]
–	GO/PES	Phase inversion	Water treatment	Water flux: 20.4 kg/m^2^/h Direct Red 16 rejection: 96% Superior anti-fouling capability	[86]

## Supplementary Materials

The following are available online at http://www.mdpi.com/2077-0375/7/3/52/s1. Figure S1: (a) GO membranes obtained from AAO filter, Teflon filter and cellulose nitrate (CN) filter had different stability in water; (b) GO (AAO) membrane remained intact whereas GO (Teflon) membrane; and (c) GO (CN) membrane readily disintegrated in water. Notes that the photos were taken after the solutions had been stirred with a lab spatula, except for the one showing GO (Teflon) and GO (CN) in water for 30 min. Figure S2: The side-view photos complementary to those shown in Figure S1 contrasting to the stability of neat GO membrane and Al^3+^ contaminated GO membrane in water. (a) The neat GO membrane (obtained with Teflon or cellulose nitrate filter paper) readily swelled upon soaking in water. After 2 h of soaking, disintegration could already be observed without any agitation. After gentle stirring with a lab spatula for a few seconds, it completely disintegrated and started to redisperse in water. In contrast, (b) the Al^3+^ crosslinked GO (AAO) membrane remained stable in water after days of soaking, which clearly demonstrated that GO (AAO) membrane was stable and neat GO membrane readily disintegrated. Figure S3: GO membrane obtained from the AAO filter was contaminated with Al^3+^. (a) Photo showed an 18 μm thick GO membrane detached from an AAO filter disc. (b) Sets of Al 2*p* spectra measured during XPS depth profiling of GO (AAO) from the bottom side and (c) from the top side. (d) Al/C ratio as a function of etching depth from both sides of the GO (AAO). (e) Depth profiling of GO (Teflon) from the bottom side suggested that no Al^3+^ was presented. Figure S4: Removal of Al^3+^ by ionic exchange. (a) Photos of GO (AAO) membrane soaked in 0.1 M HCl for 3 days (top) and then in water for 30 min (bottom); (b) Ionic exchange with monovalent cations such as Na^+^ and Li^+^ led to the removal of Al^3+^ and disintegrated of GO (AAO) membranes in water; (c) XPS Al 2*p* spectra of GO (AAO) membrane before and after HCl treatment, suggesting removal of Al^3+^. Note: All the photos were taken after the solutions were stirred with a lab spatula. Figure S5: Strengthening of GO (Teflon) membrane by Al^3+^ crosslinking. (a) Photos of GO (Teflon) membrane soaked in 0.1 M Al(NO_3_)_3_ for 1 day (top) and then in water for 5 days (bottom); (b) XRD patterns of GO (Teflon) membranes. GO (Teflon) membrane (non-crosslinked) disintegrated in water and damaged interlayer ordering. While in Al(NO_3_)_3_, it swelled and maintained the lamellar structure. After drying, the presence of Al^3+^ resulted in slightly larger interlayer spacing than that of neat GO membrane. Upon rehydration, the Al^3+^ crosslinked GO membrane swelled to yield a lamellar structure with better stacking.
