# Supplementary material for: Recent Developments of Graphene Oxide-Based Membranes: A Review
Source: Membranes (Basel). 2017 Sep 12;7(3):52. doi: 10.3390/membranes7030052 (PMC5618137; doi:10.3390/membranes7030052)
Supplement: Supplementary file 1 [file membranes-07-00052-s001.pdf]

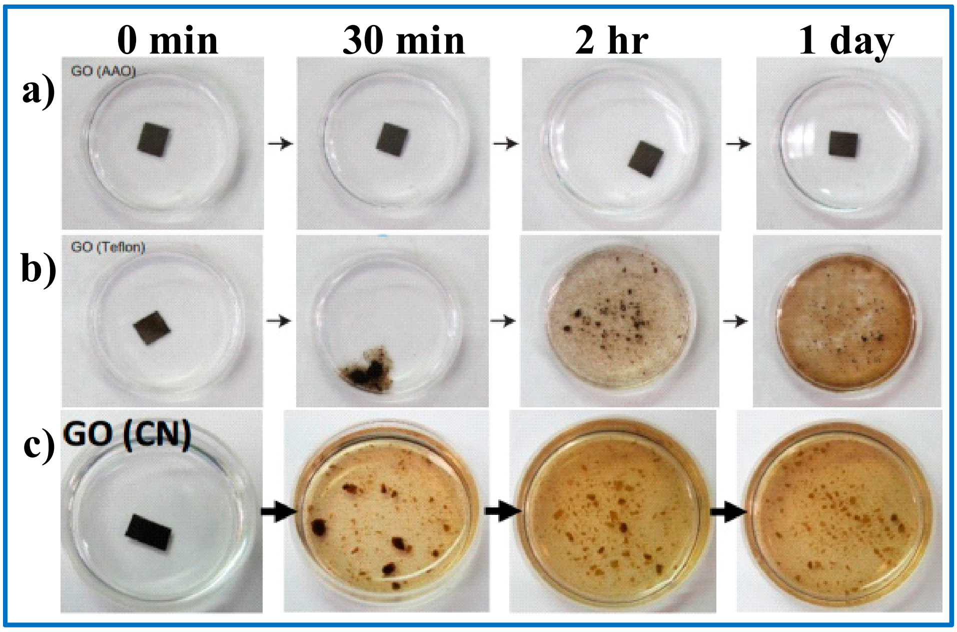

Figure S1. (**a**) GO membranes obtained from AAO filter, Teflon filter and cellulose nitrate (CN) filter had different stability in water; (**b**) GO (AAO) membrane remained intact whereas GO (Teflon) membrane; and (**c**) GO (CN) membrane readily disintegrated in water. Notes that the photos were taken after the solutions had been stirred with a lab spatula, except for the one showing GO (Teflon) and GO (CN) in water for 30 min. Copyright 2015 Nature Publishing Group.

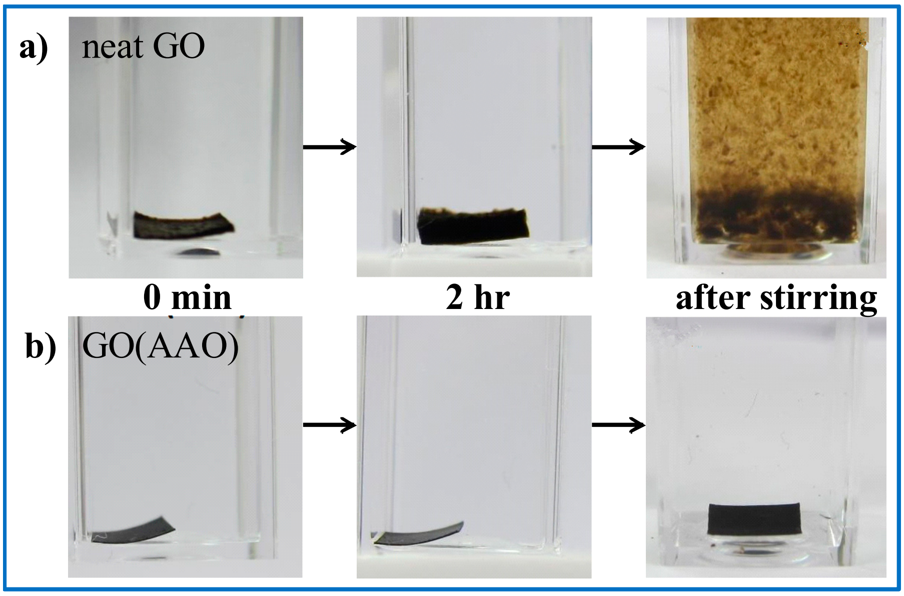

Figure S2. The side-view photos complementary to those shown in Figure S1 contrasting to the stability of neat GO membrane and Al^{3+} contaminated GO membrane in water. (**a**) The neat GO membrane (obtained with Teflon or cellulose nitrate filter paper) readily swelled upon soaking in water. After 2 h of soaking, disintegration could already be observed without any agitation. After gentle stirring with a lab spatula for a few seconds, it completely disintegrated and started to redisperse in water. In contrast, (**b**) the Al^{3+} crosslinked GO (AAO) membrane remained stable in water after days of soaking, which clearly demonstrated that GO (AAO) membrane was stable and neat GO membrane readily disintegrated. Copyright 2015 Nature Publishing Group.

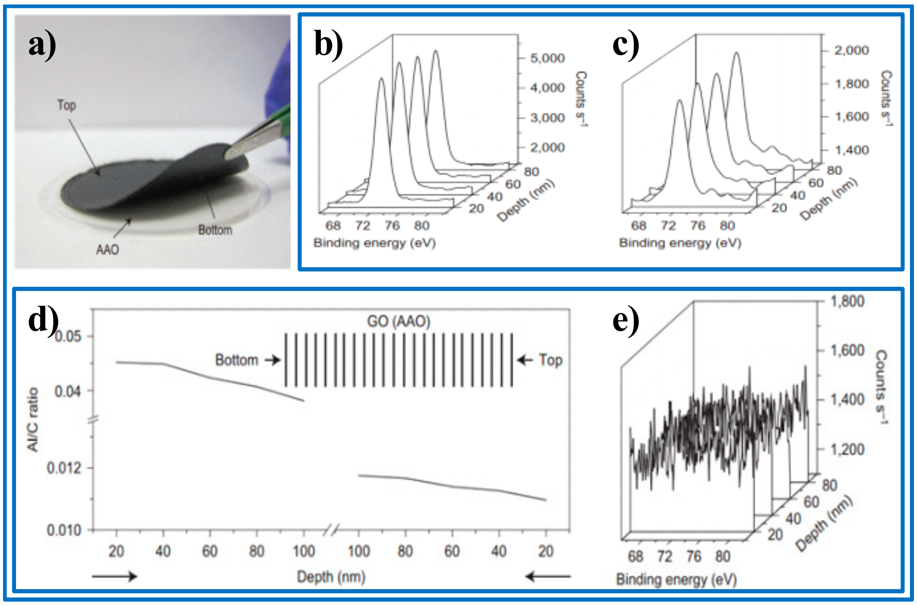

Figure S3. GO membrane obtained from the AAO filter was contaminated with Al^{3+}. (**a**) Photo showed an 18 μm thick GO membrane detached from an AAO filter disc. (**b**) Sets of Al 2*p* spectra measured during XPS depth profiling of GO (AAO) from the bottom side and (**c**) from the top side. (**d**) Al/C ratio as a function of etching depth from both sides of the GO (AAO). (**e**) Depth profiling of GO (Teflon) from the bottom side suggested that no Al^{3+} was presented. Copyright 2015 Nature Publishing Group.

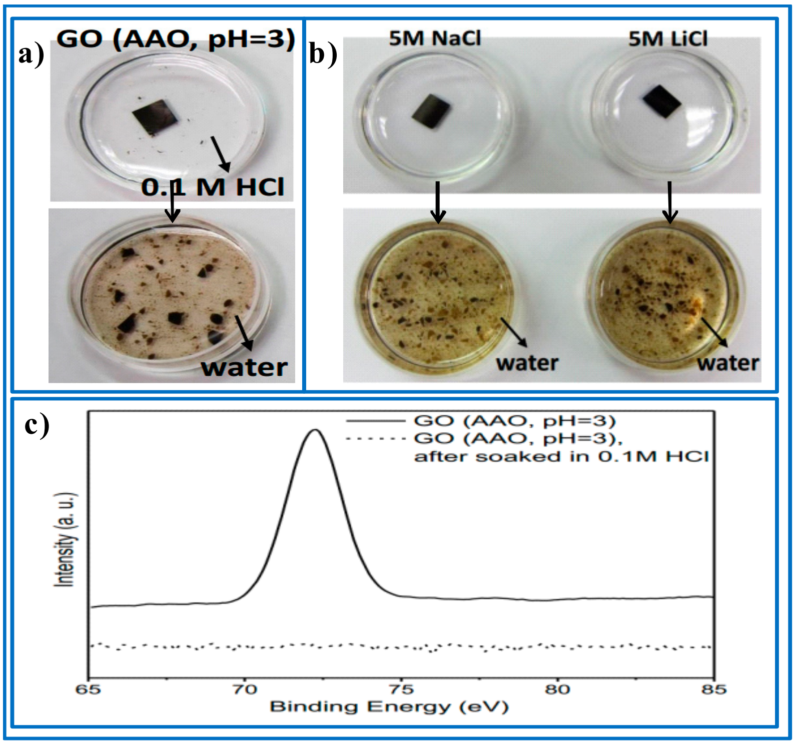

Figure S4. Removal of Al^{3+} by ionic exchange. (**a**) Photos of GO (AAO) membrane soaked in 0.1 M HCl for 3 days (top) and then in water for 30 min (bottom); (**b**) Ionic exchange with monovalent cations such as Na^+ and Li^+ led to the removal of $Al3^+$ and disintegrated of GO (AAO) membranes in water; (**c**) XPS Al 2*p* spectra of GO (AAO) membrane before and after HCl treatment, suggesting removal of Al^{3+}. Note: All the photos were taken after the solutions were stirred with a lab spatula. Copyright 2015 Nature Publishing Group.

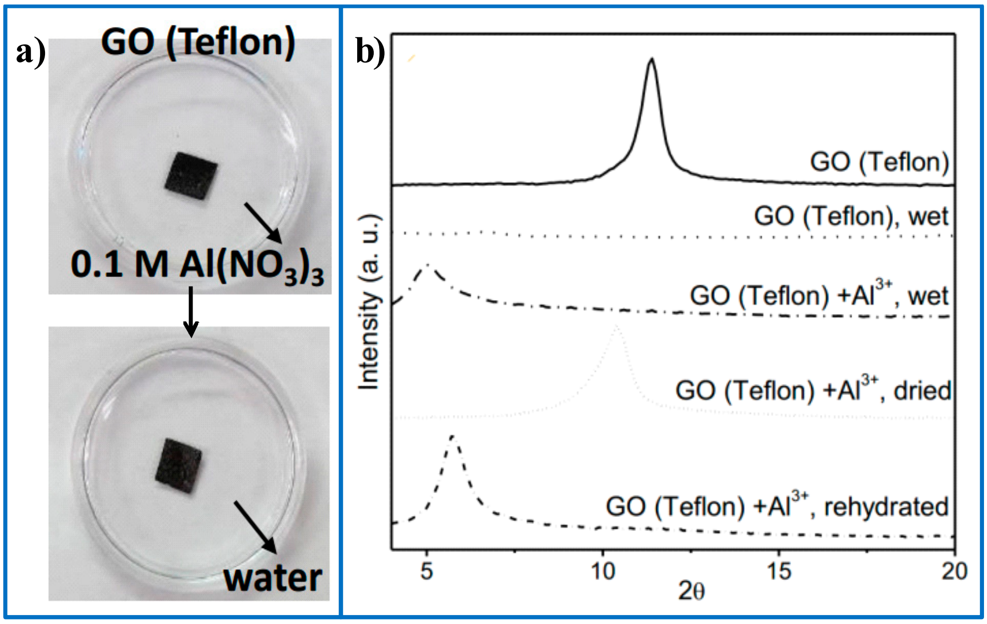

Figure S5. Strengthening of GO (Teflon) membrane by Al^{3+} crosslinking. (**a**) Photos of GO (Teflon) membrane soaked in 0.1 M $Al(NO_3)_3$ for 1 day (top) and then in water for 5 days (bottom); (**b**) XRD patterns of GO (Teflon) membranes. GO (Teflon) membrane (non-crosslinked) disintegrated in water and damaged interlayer ordering. While in $Al(NO_3)_3$, it swelled and maintained the lamellar structure. After drying, the presence of Al^{3+} resulted in slightly larger interlayer spacing than that of neat GO membrane. Upon rehydration, the Al^{3+} crosslinked GO membrane swelled to yield a lamellar structure with better stacking. Copyright 2015 Nature Publishing Group.
